# The effects of low-dose IL-2 on Th17/Treg cell imbalance in primary biliary cholangitis mouse models

**DOI:** 10.1186/s12876-024-03176-0

**Published:** 2024-02-26

**Authors:** Zilong Wang, Zhicheng Liu, Jiarui Zheng, Linxiang Huang, Rui Jin, Xiaoxiao Wang, Dongbo Chen, Yandi Xie, Bo Feng

**Affiliations:** Beijing Key Laboratory of Hepatitis C and Immunotherapy for Liver Diseases, Peking University People’s Hospital, Peking University Hepatology Institute, Beijing International Cooperation Base for Science and Technology on NAFLD Diagnosis, Beijing, China

**Keywords:** Primary biliary cholangitis, Interleukin 2, Treg cells, Th17 cells, Mouse model

## Abstract

**Background/aims:**

Primary biliary cholangitis (PBC) is a chronic cholestatic liver disease. The imbalance of Th17/Treg cells has been reported in PBC patients. Low-dose IL-2 can alleviate disease severity through modulating CD4 + T cell subsets in patients with autoimmune diseases. Hence, the present study aimed to examine the effects and mechanism of low-dose IL-2 in PBC mouse models.

**Methods:**

PBC models were induced in female C57BL/6 mice by two immunizations with 2OA-BSA at two-week intervals, and poly I: C every three days. PBC mouse models were divided into the IL-2 treated and untreated groups and low-dose IL-2 was injected at three different time points. Th17 and Tregs were analyzed by flow cytometry, and the related cytokines were analyzed by ELISA. Liver histopathology was examined by H&E and immunohistochemical staining.

**Results:**

Twelve weeks after modeling, the serum AMA was positive and the ALP was significantly increased in PBC mouse models (*P*<0.05). The pathology showed lymphocyte infiltration in the portal area, damage, and reactive proliferation of the small bile duct (*P*<0.05). The flow cytometric showed the imbalance of Th17/Treg cells in the liver of PBC mouse models, with decreased Treg cells, increased Th17 cells, and Th17/Treg ratio (*P* < 0.05). After the low-dose IL-2 intervention, biochemical index and liver pathologies showed improvement at 12 weeks. Besides, the imbalance of Th17 and Treg cells recovered. Public database mining showed that Th17 cell differentiation may contribute to poor response in PBC patients.

**Conclusion:**

Low-dose IL-2 can significantly improve liver biochemistry and pathology by reversing the imbalance of Th17 and Treg cells, suggesting that it may be a potential therapeutic target for PBC.

**Supplementary Information:**

The online version contains supplementary material available at 10.1186/s12876-024-03176-0.

## Introduction

Primary biliary cholangitis (PBC) is a chronic cholestatic liver disease characterized by progressive nonsuppurative cholangitis [1]. Without pharmacological interventions, patients can progress to liver cirrhosis and hepatocellular carcinoma. Literature reports indicate that PBC prevalence ranged from 21.7 to 39.2 per 100,000 individuals between 2004 and 2014 [2]. Ursodeoxycholic acid (UDCA) and obeticholic acid (OCA) are the approved first- and second-line treatments for patients with PBC [3], yet they have limitations, including suboptimal clinical responses and adverse effects.

The precise pathogenesis of PBC is not extremely elucidated but appears to be influenced by environmental, genetic, and immunological factors [4]. T cells play a critical role in the disease’s pathogenesis, with Th17 and Treg cells—subsets of CD4 + T cells—exhibiting a reciprocal developmental relationship. The balance between these subsets is crucial for health and disease regulation [5, 6]. Patients with PBC exhibit elevated Th17 and reduced Treg cell levels, a finding also observed in the PBC mouse model (IL-2 Rα-/- mice) [7, 8]. Hence, correcting the imbalance of Th17/Treg may be a useful target for treating PBC.

Research indicates that IL-2 modulates Th17/Treg balance by increasing Treg and decreasing Th17 cell frequencies [9]. Recently, IL-2 has been used in clinical applications and has achieved good clinical results. Clinical applications of low-dose IL-2 therapy have shown promise, demonstrating safety and effectiveness in treating systemic lupus erythematosus and influencing Th17/Treg rather than Th1/Th2 cell balance [10]. Furthermore, IL-2 has proven safe and effective in managing various autoimmune diseases, including ankylosing spondylitis (AS) and rheumatoid arthritis (RA) [11].

Although extensively studied in other autoimmune disorders, the roles of Th17 and Treg cells in PBC remain unclear. the relevance of IL-2 in Th17 and Treg cell function within the context of PBC has not been thoroughly examined. It is also unknown how IL-2 might affect disease progression and outcomes. Therefore, this study aims to investigate the dynamics of Th17 and Treg cells at different stages post-modeling and to assess the impact of IL-2 on these cell populations, as well as on the pathological and biochemical features in PBC mouse models.

## Materials and methods

### Animal experiments

Female C57BL/6 mice, aged 4–6 weeks, were purchased from Beijing Weitong Lihua Experimental Animal Technology (Beijing, China), and raised in a specific pathogen-free (SPF) facility at the Animal Center of Peking University People’s Hospital. The animal house was well-ventilated with 12-hour light/dark cycles in 25–30 °C ambient temperatures. The study was approved by the Ethics Committee of Peking University People’s Hospital (2020PHE081) and adhered to the United States Public Health Service Policy on Humane Care and Use of Laboratory Animals, as well as the ARRIVE guidelines (http://www.nc3rs.org.uk/arrive-guidelines).

PBC was induced by two immunizations with 2-nonynoic acid (2OA-BSA) at two-week intervals. The first injection was prepared with the same volume of complete Freund’s adjuvant, and the second was administered with incomplete Freund’s adjuvant, and the final concentration of the reagent was 100 µg/100µL. Polyinosinic polycytidylic acid (poly I: C) (5 mg/kg) was injected every three days via subcutaneous injections (Supplementary Fig. S1) [12]. Control mice received phosphate-buffered saline (PBS) through the same routes at corresponding time points.

For the treatment assessment, PBC mouse models were allocated to either the treated or untreated groups. The treated group received subcutaneous injections of low-dose IL-2 (30,000 IU) (Supplementary Fig. S2) every three days at three specified time points: three days before modeling, and four and eight weeks post-modeling. The untreated group received saline injections following the same schedule (Supplementary Fig. S3).

### Flow cytometry

Flow cytometric analysis was conducted on mononuclear cells (MNCs) isolated from the liver. Livers were removed and single-cell suspensions were prepared and purified from 40/70% Percoll (GE Healthcare) interphase after discontinuous Percoll gradient. Cell suspensions with 1 × 10^6^ cells were stained in dark. After surface staining of CD3, CD4, and CD25 and permeabilization/fixation, cells were stained for intracytoplasmic IL-17 A and intranuclear Foxp3. In addition, the levels of IL-17 A were detected after in vitro activation with a cell activation cocktail and blocking with brefeldin. Th17 cells were defined as CD3^+^CD4^+^IL-17 A^+^ and Treg cells were defined as CD3^+^CD4^+^ Foxp3^+^ (Supplementary Fig. S4, Table. S1).

### H&E and immunohistochemistry

For Hematoxylin and eosin (H&E) staining, liver sections were paraffin-embedded, deparaffinized, rehydrated, and stained using standard protocols. Imaging was performed using an inverted microscope (Olympus, U-LH75XEAPO). Liver pathological scores were evaluated according to the scoring system adapted from the previous literature by two pathologists [13]. The pathological scoring ranges from zero to eleven, including biliary duct involvement (0–3), biliary epithelial proliferation (0–4), and mononuclear leucocytic infiltration (0–4).

Immunohistochemistry followed similar initial steps as H&E staining. After quenching endogenous peroxidase activity, sections were incubated with primary antibodies (CD4, CD8, CK-19, Rorγ) (Supplementary Fig. S4, Table. S2). overnight at 4 °C, followed by a 30-minute incubation with horseradish peroxidase-conjugated secondary antibodies at 37 °C. Post-washing with PBS, diaminobenzidine (DAB) was applied for visualization. Microscopic examination was conducted with a light microscope (Olympus).

### Enzyme-linked immunosorbent assay

The levels of IL-17 A, IL-10, TGF-β, and PDC-E2 antibodies (ELISA kits are listed in Supplementary Table S3) in the mouse serum were measured by ELISA. The operations were conducted strictly according to the kit instructions. In brief, the diluted detection antibody was added to diluted sera and incubated at room temperature for 1.5 h. After plates were washed 5 times, horseradish peroxidase (HRP)-conjugated streptavidin was added to each well followed by incubation at room temperature. After incubation, the substrate solution was added and measured at 450 nm. The concentration of in ELISA was calculated by the standard curve derived from the standard sample provided in ELISA kit.

### Data sources and differentially expressed gene (DEGs) analysis

The GEO database (https://www.ncbi.nlm.nih.gov/geo/) is an open-source and public platform for the storage of genetic data. Liver RNA-sequencing data (GSE79850) from PBC patients were published previously and was available for download from the GEO website, including UDCA responders (*n* = 7), UDCA non-responders (*n* = 9) and non-liver disease controls (*n* = 9).

In the GSE79850 dataset, we analyzed DECs by using the “limma” package in the software R 4.1.2 (Vienna, Ausria). DEGs were defined as the absolute value of log2FC ≥ 1 as well as the false discovery rate (FDR) < 0.05 [14]. Gene ontology (GO) and pathway enrichment analysis (Kyoto Encyclopedia of Genes and Genomes (KEGG)) were implemented using the “ClusterProfiler” package, and the threshold for significance was a *p*-value less than 0.05.

### Statistical analysis

Data were expressed as mean ± standard deviation and were analyzed using student t-tests or one-way ANOVA by GraphPad Prism 7 software (GraphPad Software Inc., San Diego, CA, USA). A *P*-value < 0.05 denoted statistical significance.

## Results

### General conditions and liver pathologies of the PBC mouse model

**Fig. 1:**
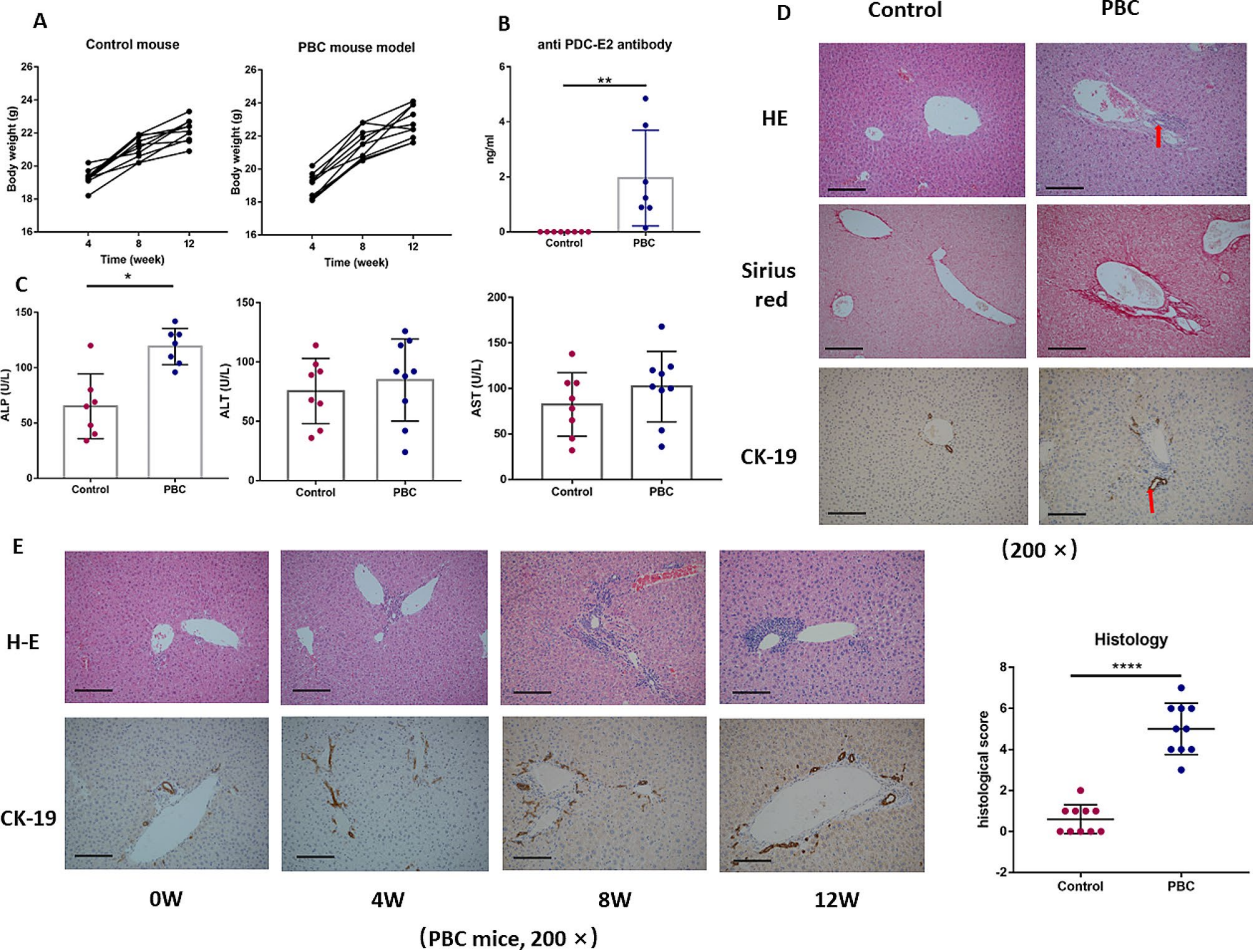
The overall performance of the PBC mouse models. (**A**) Comparative body weight trajectory in control and PBC mouse models over a period of 4–12 weeks post-model induction. (**B**) ELISA-based quantification of anti-PDC-E2 antibodies in the sera of control and PBC mouse models. (**C**) Biochemical analysis of serum parameters in control and PBC mouse models. (**D**) Hepatic histopathology visualized with H&E staining and CK-19 immunohistochemistry highlighting cholangiocytes. (**E**) Temporal progression of liver pathology in PBC mouse models post-model induction

Throughout the modeling process, no significant differences were observed in the general condition of the mice, including eating, drinking, body weight, activity, and coat color (Fig. 1A). No signs of jaundice were observed in the PBC mouse model. The serum anti-PDC-E2 could be detected in the PBC mouse model (Fig. 1B). Besides, Alkaline phosphatase (ALP) levels were significantly elevated in the PBC model compared to controls (119.10 ± 6.20 vs. 64.67 ± 27.72 U/L, *P*<0.05). However, no notable differences in alanine aminotransferase (ALT) (72.67 ± 13.77 vs. 72.6 ± 22.64 U/L, *P*>0.05) and aspartate aminotransferase (AST) (118.3 ± 9.16 vs. 116.7 ± 10.67 U/L, *P*>0.05) levels were found between the groups. (Fig. 1C).

After injection of 2OA-BSA and poly I: C, prominent inflammatory infiltration, extensive bile duct destruction, and significant bile duct proliferation were apparent in the PBC model (Fig. 1D). These pathological changes became more severe as the modeling duration increased, with a marked difference in liver pathology scores between the PBC and control mice (0.6 ± 0.70 vs. 55.0 ± 1.25, *P*<0.05, Fig. 1E).

### The disturbance of Th17/Treg balance in the liver in the PBC mouse model

**Fig. 2:**
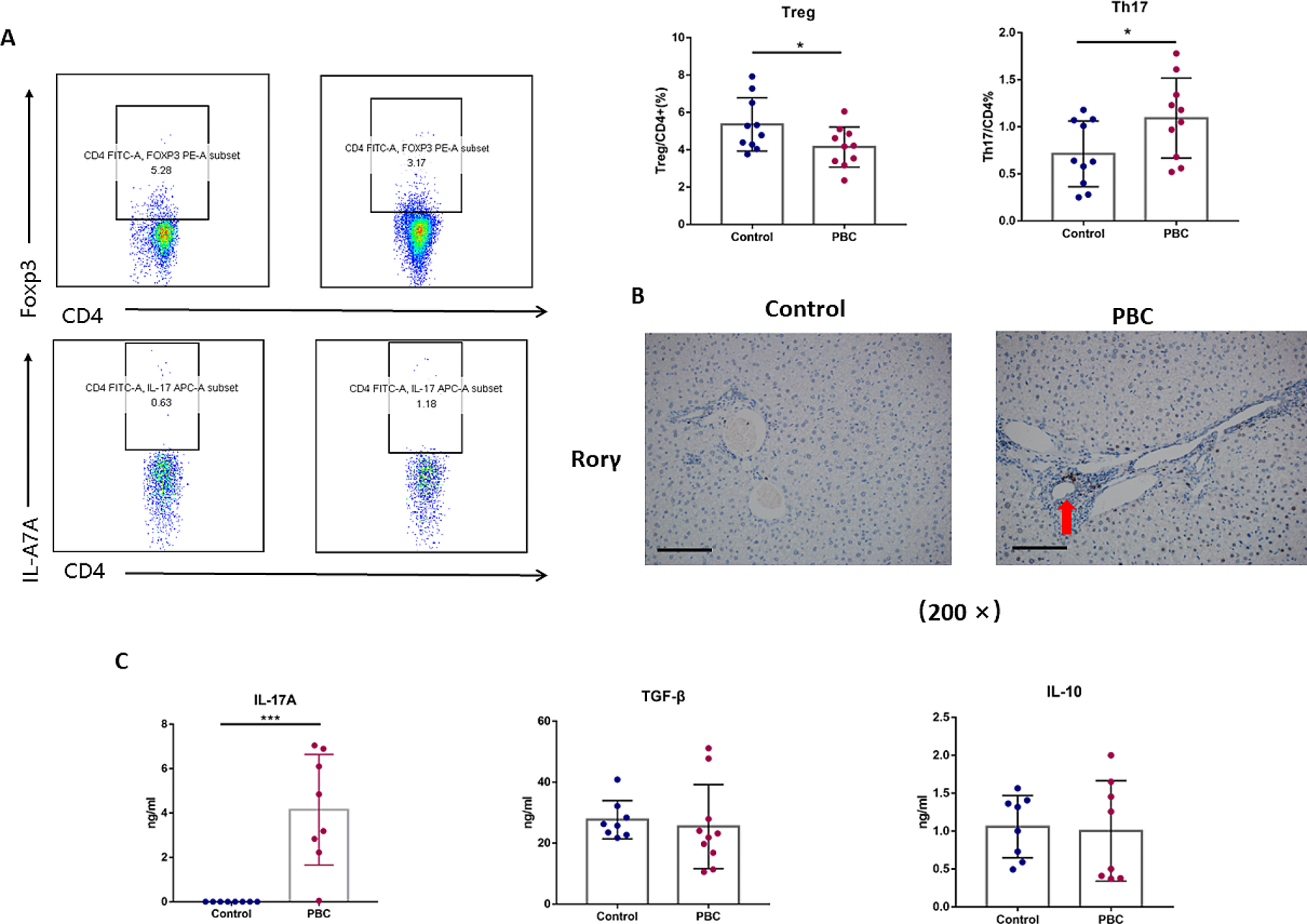
The imbalance of Th17 and Treg has an important role in PBC mouse models. (**A**) Flow cytometric analysis revealing augmented Th17 and diminished Treg cell populations in PBC mouse livers. (**B**) Histological evidence of Th17 cell infiltration (red arrows) in PBC mouse livers (magnification: ×200). (**C**) Comparative serum profiling of Th17- and Treg-associated cytokines between control and PBC mouse models

Flow cytometry revealed an increase in Th17 cell populations and a decrease in Treg cells within the livers of PBC mouse models compared to controls (0.38 ± 0.05 vs. 0.84 ± 0.09%, *P* < 0.05 for Th17; 5.70 ± 0.53 vs. 4.32 ± 0.29%, *P* < 0.05 for Treg). (Fig. 2A). The imbalance between Th17 and Treg cells worsened over time. Immunohistochemical staining indicated Th17 cell infiltration around bile duct epithelial cells in the portal tracts, correlating with increased hepatic inflammation and suggesting a role in exacerbating bile duct damage. (Fig. 2B).

Additionally, serum cytokine levels measured by ELISA revealed a significant rise in IL-17 A in PBC mouse models (0 vs. 4.55 ± 0.60 ng/ml, *P* < 0.01). There was also a downward trend in Treg-related cytokines TGF-β (51.78 ± 0.89 vs. 36.48 ± 13.00 ng/ml, *P* = 0.19) and IL-10 (0.89 ± 0.02 vs. 0.97 ± 0.02 ng/ml, *P* = 0.49), though not statistically significant. (Fig. 2C). The above results suggested that the level of Th17- and Treg-related cytokines have the same changing trend as Th17 and Treg cells.

### Biochemical and pathological changes after low-dose IL-2-treated in PBC mouse model

**Fig. 3:**
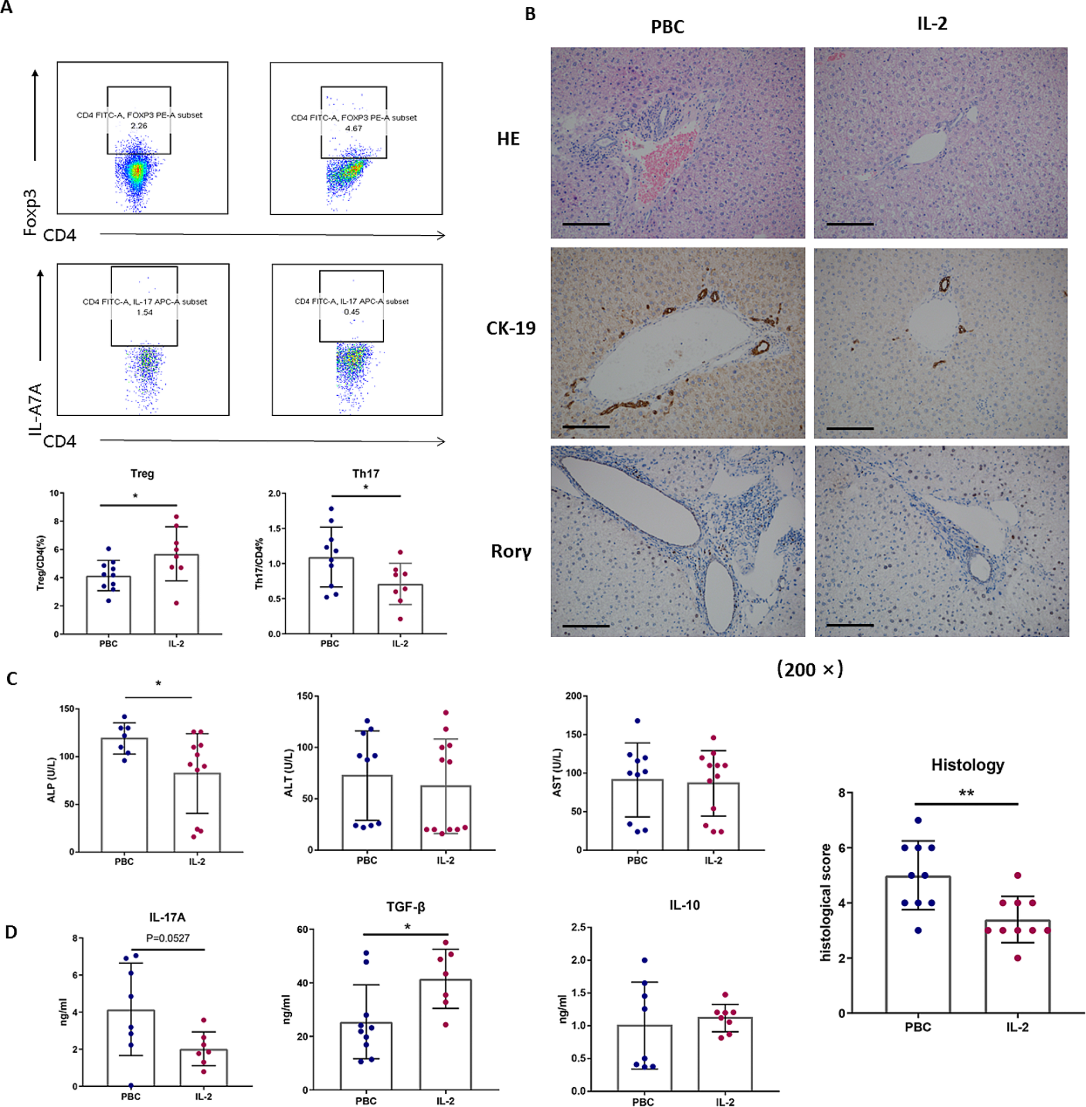
Immunological and Pathological Response to Low-Dose IL-2 Therapy in PBC Mouse Models. (**A**) Flow cytometric evaluation indicating a reduction in Th17 and an elevation in Treg populations within PBC mouse livers post low-dose IL-2 treatment. (**B**) H&E staining and CK-19 immunohistochemistry of liver sections post low-dose IL-2 treatment. Th17 cell hepatic infiltration following low-dose IL-2 therapy in PBC mouse models (magnification: ×200). (**C**) Biochemical serum parameter analysis post low-dose IL-2 intervention in control and PBC mouse models. (**D**) Serum Th17- and Treg-related cytokine levels following low-dose IL-2 therapy in control versus PBC mouse models

Four weeks post-initiation of modeling, mice were administered low-dose IL-2 treatment until the twelfth week. Subsequent flow cytometry analysis revealed an increased infiltration of Treg cells and a decrease in Th17 cells (*P* < 0.05), suggesting a restoration of the Th17/Treg cell balance (Fig. 3A). The intervention attenuated inflammatory infiltration within the portal area, diminished bile duct damage, and inhibited ductular proliferation, as evidenced by significantly lower liver pathology scores in treated mice (6.00 ± 1.00 vs. 2.50 ± 0.50, *P* < 0.05). Immunohistochemical analysis further confirmed a reduction in Th17 cell infiltration in the portal areas (Fig. 3B). Notably, alkaline phosphatase (ALP) levels significantly decreased in the treated group compared to the untreated PBC mouse models (119.10 ± 6.20 vs. 82.36 ± 12.50 U/L, *P* < 0.05), while alanine aminotransferase (ALT) and aspartate aminotransferase (AST) levels remained unaltered (Fig. 3C). Moreover, enzyme-linked immunosorbent assay (ELISA) indicated a significant reduction in serum IL-17 A and an increase in TGF-β levels in the IL-2-treated mouse (4.55 ± 0.60 vs. 19.28 ± 0.39 ng/ml, *P* = 0.053 for IL-17 A; 25.45 ± 13.79 vs. 41.48 ± 11.04 ng/ml, *P* < 0.05 for TGF-β).

### The effect of low-dose IL-2 at different points differed in each group

**Fig. 4:**
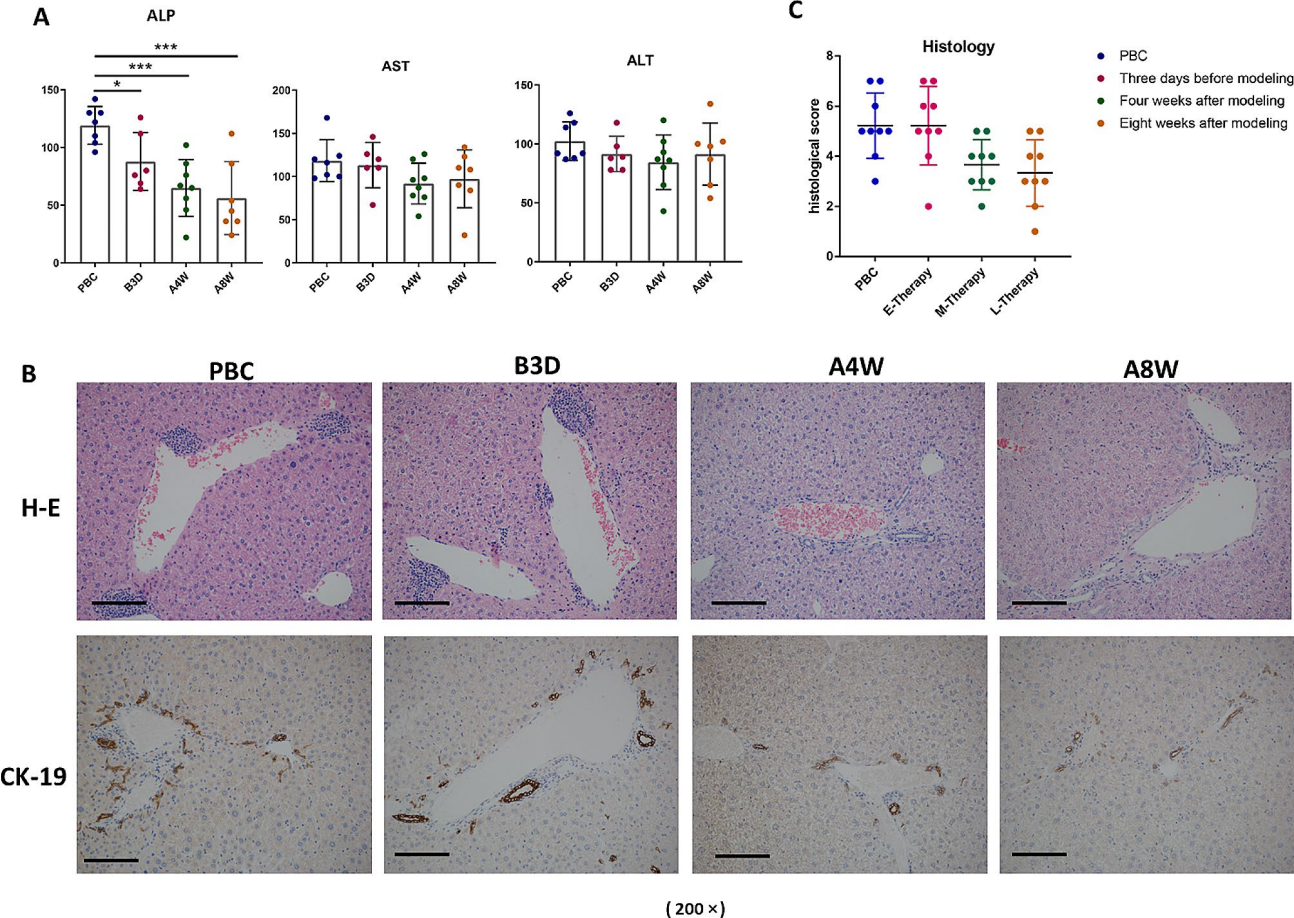
Temporal Analysis of Low-Dose IL-2 Therapy Effects in PBC Mouse Models. (**A**) Biochemical serum parameter analysis at various time points during low-dose IL-2 therapy (three days pre-modeling, four and eight weeks post-modeling). (**B**) Histopathological comparison of liver sections stained with H&E and CK-19 antibodies across treatment groups. (**C**) Mean liver pathology scoring across various treatment groups. B3D: Three days before modeling. A4W: Four weeks after modeling. A8W: Eight weeks after modeling

When comparing the effect of low-dose IL-2 administered at different time points, including three days prior to modeling, and four and eight weeks post-modeling, the pre-treated group showed less improvement in ALP levels (94.50 ± 14.64 vs. 68.40 ± 13.79 vs. 62.40 ± 16.22 U/L, *P* < 0.05) (Fig. 4A), and in portal inflammation and bile duct pathology (5.22 ± 0.52 vs. 3.67 ± 0.33 vs. 3.33 ± 0.44, *P* < 0.05) (Fig. 4B, C). This suggests that mice receiving treatment before disease induction experienced limited benefit compared to those treated post-modeling.

### Increased Th17 cell populations were associated with UDCA suboptimal response in PBC patients

The GSE79850 dataset was obtained from GEO database, including 8 non-liver disease patients and 16 PBC patients (7 UDCA responders and 9 UDCA non-responders). We then compared the key transcription factor RORC of Th17 expression in all three groups after standardization. It was found that the RORC expression level was notably increased in the UDCA responder group (6.36 ± 0.42 vs. 8.49 ± 0.10, *P*<0.01) compared to control health, suggesting that Th17 cell populations were increased in liver tissue of patients with PBC.

**Fig. 5:**
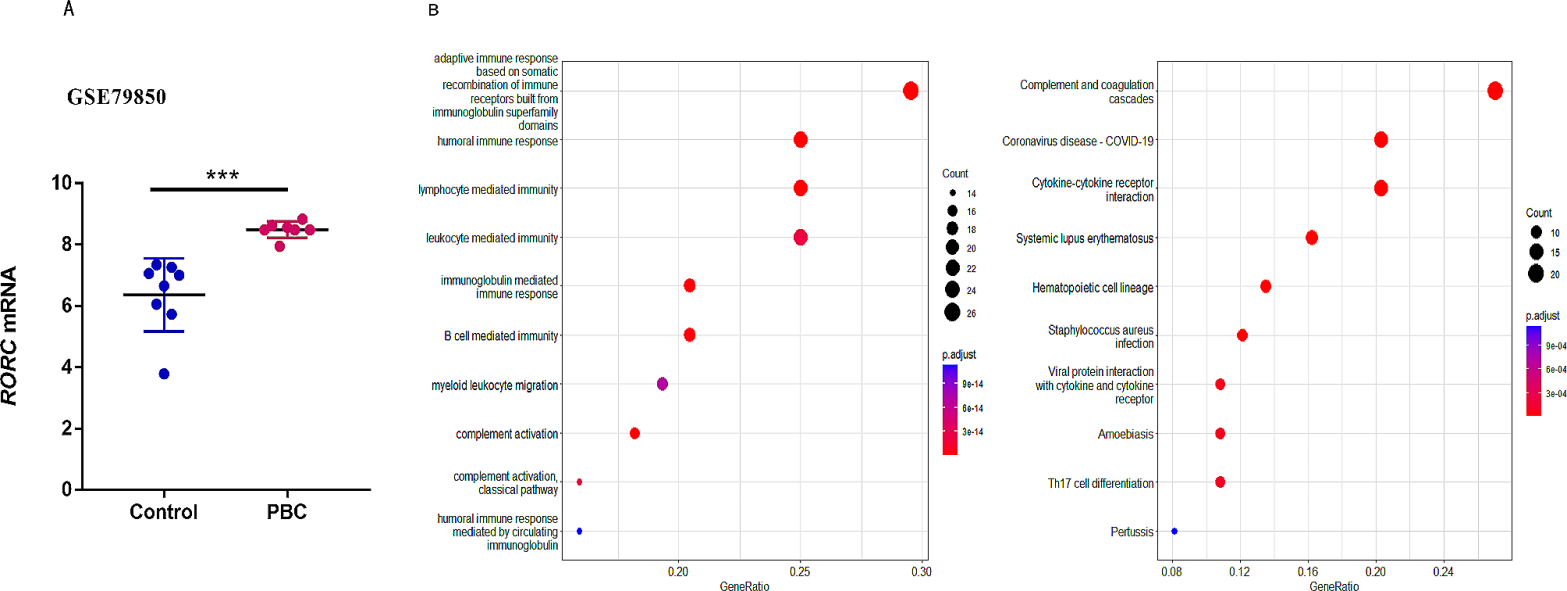
Bioinformatics analysis of DEGs for PBC patients in a public database. (**A**) Increased Th17 cell prevalence in PBC patient (UDCA response well) livers according to dataset GSE79850. (**B**) GO and KEGG enrichment analysis of differential genes in the UDCA response well and poorly groups, and the main enrichment pathways were immune related pathways and Th17 cell differentiation. GO: Gene Ontology; KEGG: Kyoto Encyclopedia of Genes and Genomes

To further elucidate the potential mechanisms of response status, a functional enrichment analysis (GO and KEGG) of differential genes was performed between UDCA responders and non-responders. GO analysis showed that among the 10 signal pathways enriched with more than 2 times of DEGs, adaptive immune response, immune receptor recombination constructed by immunoglobulin superfamily domain, humoral immunity, lymphocyte-mediated immunity, and other pathways were related to immunity, suggesting that immune factors may be a vital factor of poor UDCA response. Moreover, KEGG enrichment pathway analysis showed that the DEGs were significantly enriched in the Th17 cell differentiation (Fig. 5B).

## Discussion

In this study, we found that PBC mouse models exhibited increased Th17 cell frequencies and decreased Treg cell frequencies. With the extension of modeling time, this trend is more pronounced. However, low-dose IL-2 treatment expanded Treg populations and diminished Th17 populations, ameliorating pathological changes and normalizing serum biochemical markers. Additionally, Th17 cell accumulation in the livers of PBC patients was observed using public databases. Differential gene expression analysis between UDCA response in well and poorly patients revealed genes associated with immune pathways, suggesting that immune regulation may be critical for enhancing treatment outcomes in PBC. The liver, a complex organ with a distinct immune profile, serves roles in phagocytosis, defense, and immune regulation. PBC involves the nonsuppurative destruction of small bile ducts, with immune responses playing a significant role in this process [15]. Single-cell RNA sequencing (scRNA-seq) and spatial transcriptomics revealed that immune cells, especially T cells, exhibited the most prominent changes in PBC patients [16].

Th17, a pro-inflammatory T helper cell subset, and Th17-related cytokines are implicated in various diseases, including autoimmune and viral liver diseases [17, 18]. Conversely, Treg cells are essential in suppressing autoimmune reactions and curbing chronic inflammation [19]. Th17 and Treg cells, though functionally divergent, have closely linked differentiation processes. In the presence of IL-6 and low TGF-β concentrations, naïve CD4 + T cells differentiate into Th17 cells, suppressing Foxp3 expression, while high TGF-β levels promote Treg differentiation [20]. Autoimmune diseases often feature a disrupted Th17/Treg balance, characterized by an increase in Th17 cells and a decrease in Treg activity [21]. Recognizing the pivotal role of Th17/Treg imbalance in PBC pathogenesis, modulation of these cells has emerged as a therapeutic strategy [22]. In this view, regulating the imbalance of Th17/Treg cells was identified as a potential therapeutic target for diseases. Arsenic trioxide [23], hypoxia-inducible factor (HIF-1α) [24], IL-21 [25], zinc [26], and other drugs have been proven to reverse the imbalance of Th17/Treg cells, playing a beneficial role in improving different disease models.

Low-dose IL-2 is acknowledged as an effective modulator of the Th17/Treg balance [10]. Studies have shown that IL-2 regulates the expression of STAT3 and STAT5, influencing Th17 and Treg cell differentiation [27]. The efficacy and safety of low-dose IL-2 have been confirmed in clinical studies involving rheumatoid arthritis [28], Primary Sjögren Syndrome [29], and systemic lupus erythematosus patients [30]. While IL-2’s benefits for autoimmune liver disease remain to be fully explored, animal models of autoimmune hepatitis and primary sclerosing cholangitis have indicated its potential to improve biochemical markers and reduce bile duct damage [31, 32]. Notably, IL-2 dosage is crucial, as low doses target Treg and Th17 regulation, whereas high doses can activate effector T cells and enhance immune responses. Therefore, determining the appropriate IL-2 dosage is essential [33].

Furthermore, the timing of administration emerged as a critical factor. the timing of administration emerged as a critical factor. The pre-treated group showed more limited improvement compared to the group treated after disease onset. Previous animal studies suggest that therapeutic use of IL-22 seems to reduces the portal inflammatory response and improve portal pathology, though positive effects have also been shown when prophylactic use of IL-22 [34]. The distinct outcomes of low-dose IL-2 administered at different stages may result from its preventive use, potentially stimulating T cell proliferation and activation, thus priming the mouse immune system. The “double hit” of IL-2 and modeling reagents on the immune system may lead to poor improvement of the PBC mouse model by preventive administration. The distinct outcomes of low-dose IL-2 administered at different stages may result from its preventive use, potentially stimulating T cell proliferation and activation, thus priming the mouse immune system.

This study has several limitations that warrant acknowledgment. Firstly, no mouse model can fully replicate the human PBC condition; thus, the liver immune environment and pathology in these models differ significantly from those in PBC patients. Secondly, IL-2’s multifaceted role in various immune cells necessitates further elucidation. Lastly, the application of low-dose IL-2 in human subjects has yet to be assessed; consequently, future clinical trials are imperative to ascertain its therapeutic potential in PBC patients.

## Conclusion

In conclusion, the dysregulation of Th17 and Treg cells plays a role in PBC pathogenesis. Low dose IL-2 may mitigate the onset and progression of PBC by rectifying the Th17/Treg imbalance and significantly enhancing liver biochemistry and pathology, indicating its potential as a therapeutic agent for PBC.

### Electronic supplementary material

Below is the link to the electronic supplementary material.

Supplementary Material 1

## Data Availability

The data that support the findings of this study are available on request from the corresponding author, who received permission from Ethics Committee of Peking University People’s Hospital.

## Abbreviations

PBC: Primary biliary cholangitis
UDCA: Ursodeoxycholic acid
OCA: Obeticholic acid
2-OA: 2-nonynoic acid
PSC: Primary sclerosing cholangitis
