# Supplementary material for: The effects of low-dose IL-2 on Th17/Treg cell imbalance in primary biliary cholangitis mouse models
Source: BMC Gastroenterol. 2024 Feb 26;24:87. doi: 10.1186/s12876-024-03176-0 (PMC10895794; doi:10.1186/s12876-024-03176-0)
Supplement: Supplementary file 1 — Supplementary Material 1 [file 12876_2024_3176_MOESM1_ESM.docx]

Supplementary Material

This file includes:

Fig.S1. The detailed process of the primary biliary cholangitis mouse model.

Fig.S2. The liver pathologies and immune cell infiltrate in the liver of mice treated with different doses of IL-2.

Fig.S3. Study design.

Fig.S4. Representative gating of Tregs and Th17 cells.

Table.S1 Antibodies Used in Flow Cytometric Analysis.

Table.S2 Antibodies Used immunohistochemical.

Table.S3 Mouse ELISA kit used in this study.


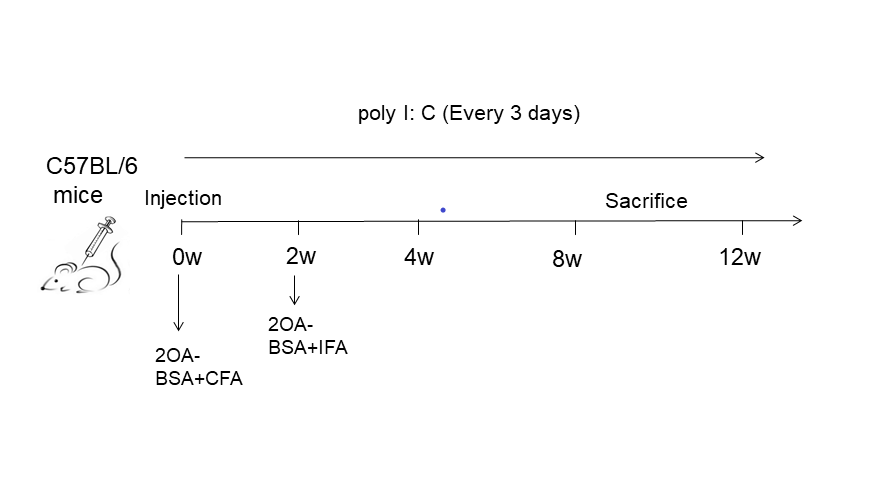


Fig.S1. The detailed process of the primary biliary cholangitis mouse model.

Primary biliary cholangitis (PBC) was elicited by two intraperitoneal immunizations with 2-nonynoic acid-bovine serum albumin (2OA-BSA) conjugate at a concentration of 100 mg/100 ml in phosphate-buffered saline (PBS), at two-week intervals. The first immunization was supplemented with an equal volume of complete Freund's adjuvant (CFA) containing 1 mg/ml Mycobacterium tuberculosis H37Ra, while the second immunization utilized 2OA-BSA with incomplete Freund's adjuvant (IFA). Additionally, polyinosinic:polycytidylic acid (poly I:C) was administered intraperitoneally at a dose of 5 mg/kg every three days, starting from three days post-initial immunization. Control female C57BL/6 mice received PBS following the same protocol.


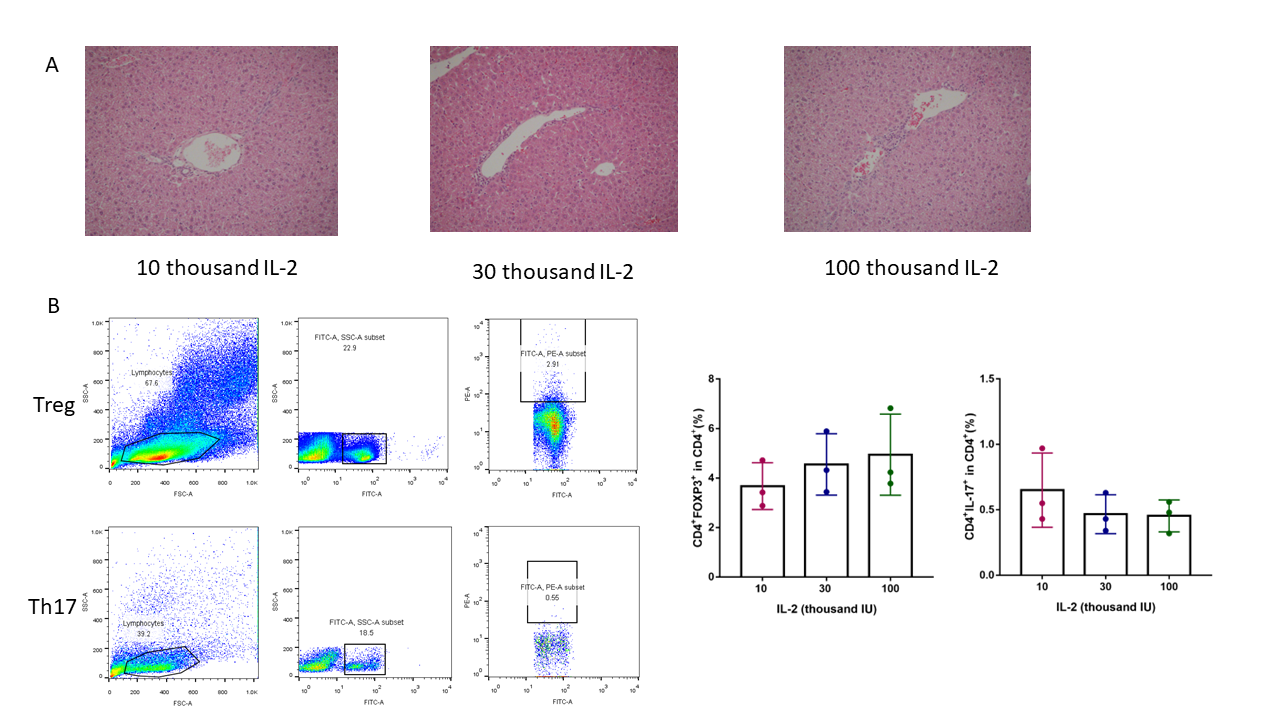


Fig.S2. The liver pathologies and immune cell infiltrate in the liver of mice treated with different doses of IL-2.

In our study, C57BL/6 mice were treated with IL-2 at doses of 10,000 IU, 30,000 IU, and 100,000 IU to assess the effects on Th17/Treg balance and liver pathology. The 10,000 IU dose did not significantly influence Treg or Th17 cells. Both the 30,000 IU and 100,000 IU doses regulated immune cells comparably; however, the 100,000 IU dose resulted in inflammatory cell infiltration in the liver.


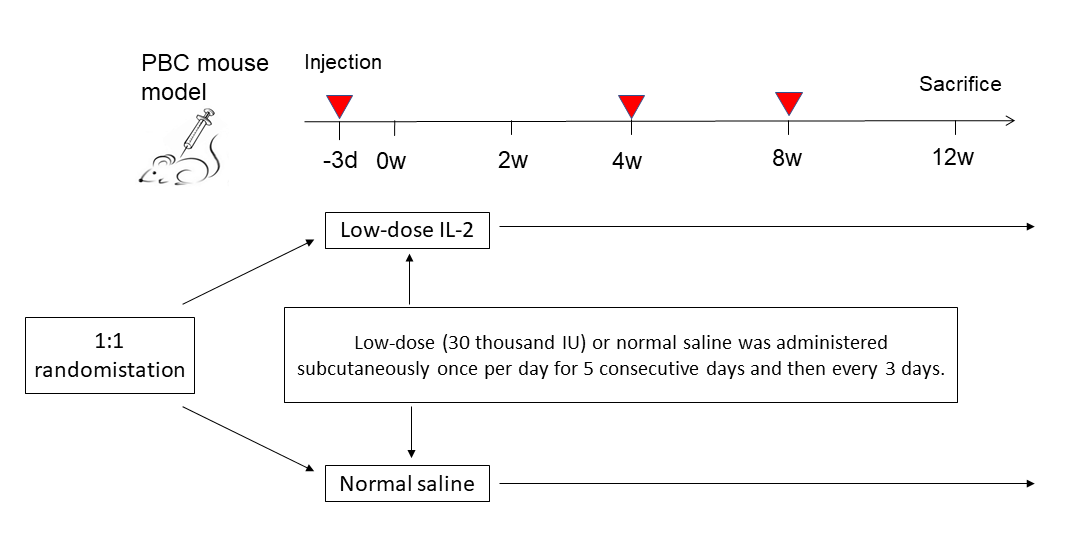


Fig.S3. Study design

For the intervention study, PBC mice were stratified into IL-2 treated and untreated cohorts. Low-dose IL-2 (30,000 IU) was administered subcutaneously daily for five consecutive days, followed by dosing every three days at three specific time points: three days prior to modeling, and at four and eight weeks post-modeling.


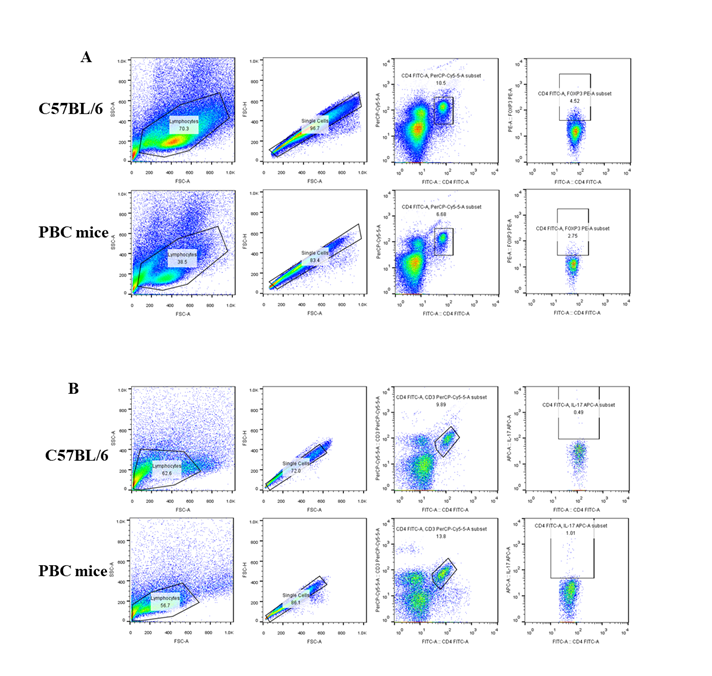


Fig.S4. Representative gating of Tregs and Th17 cells.

Table.S1 Antibodies Used in Flow Cytometric Analysis.

| Target antibody | Fluorochrome | Brand | Cat No |
| --- | --- | --- | --- |
| CD3 | FITC | Biolegend | 300306 |
| CD4 | PerCp | Biolegend | 317432 |
| Foxp3 | PE | Biolegend | 100326 |
| IL-17A | APC | Biolegend | 506916 |

Table.S2 Antibodies Used immunohistochemical.

Table.S2 Antibodies Used immunohistochemical.

| Target antibody | Brand | Cat No |
| --- | --- | --- |
| CD4 | Abcam | Ab183685 |
| CD8 | Abcam | Ab21769 |
| CK-19 | Abcam | Ab52625 |
| Rorgama | Abcam | Ab204082 |

Table.S3 Mouse ELISA kit used in this study.

| Target antibody | Brand | Cat No |
| --- | --- | --- |
| PDC-E2 | MEIMIAN | MM-46349M1 |
| IL-17A | MULTI SCIENCES | EK217 |
| TGF-β | MULTI SCIENCES | EK981 |
| IL-10 | MULTI SCIENCES | EK210 |
